# Validation of a new tool to assess health-related quality of life in psoriasis: the PSO-LIFE questionnaire

**DOI:** 10.1186/1477-7525-10-56

**Published:** 2012-05-24

**Authors:** Esteban Dauden, Enrique Herrera, Lluis Puig, José Luis Sánchez-Carazo, Jaime Toribio, Ma Teresa Caloto, Gonzalo Nocea, Montse Roset, Nuria Lara

**Affiliations:** 1 Department of Dermatology, Hospital Universitario de la Princesa, Madrid, Spain; 2 Department of Dermatology, Hospital Universitario Virgen de la Victoria, Málaga, Spain; 3 Department of Dermatology, Hospital de la Santa Creu i Sant Pau, Barcelona, Spain; 4 Department of Dermatology, Hospital General de Valencia, Valencia, Spain; 5 Department of Dermatology, CHU Santiago (Hospital Gil Casares), Santiago de Compostela, Spain; 6 Outcomes Research, MSD, Madrid, Spain; 7 IMS Health, Health Economics and Outcomes Research, Barcelona, Spain

**Keywords:** Psoriasis, Quality of life, Questionnaire, Validation

## Abstract

**Background:**

Several questionnaires have been used to measure health related quality of life (HRQoL) in patients with psoriasis, few have been adapted for use in Spain; none of them was developed specifically for the Spanish population. The purpose of the study was to validate and assess the sensitivity to change of a new questionnaire to measure HRQOL in patients with psoriasis (PSO-LIFE).

**Methods:**

Observational, prospective, multicenter study performed in centers around Spain. Patients with active or inactive psoriasis completed the PSO-LIFE together with other Dermatology Quality of Life Index (DLQI) and Psoriasis Disability Index (PDI). A control group of patients with urticaria or atopic dermatitis was also included. Internal consistency and test-retest reliability of the PSO-LIFE were assessed by calculating Cronbach’s alpha and Intraclass Correlation Coefficient (ICC). Validity was assessed by examining factorial structure, the capacity to discriminate between groups, and correlations with other measures. Sensitivity to change was measured using effect sizes.

**Results:**

The final sample included for analysis consisted of 304 patients and 56 controls. Mean (SD) age of psoriasis patients was 45.3 (14.5) years compared to 38.8 (14) years for controls (p < 0.01). Cronbach’s alpha for the PSO-LIFE was 0.95 and test-retest reliability using the ICC was 0.98. Factor analysis showed the questionnaire to be unidimensional. Mean (SD) PSO-LIFE scores differed between patients with psoriasis and controls (64.9 [22.5] vs 69.4 [17.3]; p < 0.05), between those with active and inactive disease (57.4 [20.4] vs 76.4 [20.6]; p < 0.01), and between those with visible and non-visible lesions (63.0 [21.9] vs. 74.8 [23.9]; p < 0.01). The correlation between PSO-LIFE and PASI scores was moderate (r = −0.43) while correlations with DLQI and PDI dimensions ranged from moderate to high (between 0.4 and 0.8). Effect size on the PSO-LIFE in patients reporting ‘much improved’ health status at study completion was 1.01 (large effect size).

**Conclusions:**

The present results provide substantial support for the reliability, validity, and responsiveness of the PSO-LIFE questionnaire in the population for which it was designed.

## Background

A number of studies have shown that psoriasis has a marked negative impact on patient’s health related quality of life (HRQoL) as well as on the quality of life of family members and partners of patients with psoriasis [1]. A study by the National Psoriasis Foundation found that 75% of patients reported that the disease had a negative impact on the daily lives [2]. A review of 17 studies of HRQoL in psoriasis showed that patients with the disease reported physical discomfort, impaired emotional functioning, a negative body and self-image, and limitations in daily activities, social contacts and (skin-exposing) activities, and work [3]. Other studies have shown that the disease has a particularly negative effect on self-perception of body image leading to low self-esteem, stigma, and a feeling of shame in the patient because of the lesions produced by the psoriasis [2]. Other areas affected are sexual and personal relations due to patients’ acute sense of shame stemming from the effect of lesions on physical appearance [4].

Several questionnaires have been used to measure HRQOL in patients with psoriasis, including generic instruments, generic instruments for dermatology and disease-specific questionnaires [5][6]. The Dermatology Life Quality Index (DLQI) [7] and the Skindex 29 [8], generic questionnaires for dermatology, have been used for psoriasis. As well as other disease specific questionnaires like the Psoriasis Disability Index (PDI) [9], the Impact of Psoriasis questionnaire (IPSO) [10], the 12-Item Psoriasis Quality of Life Questionnaire (PQoL-12) [11], the Psoriasis Index of Quality of Life (PSORIQOL) [12], the Psoriasis Life Stress Inventory (PLSI) [13], and the Questionnaire on Experience with Skin Complaints (SF-QES) [14].

Of these, only the DLQI and the PDI have been adapted for use in Spain [15][16]. In psoriasis patients, the DLQI has been shown to have a notable floor effect and a lack of sensitivity to detect changes when clinically significant changes occur [1]. Likewise, the PDI concentrates largely on symptoms and only to a limited degree on the impact of the disease on patients’ QOL. None of these questionnaires was developed specifically for the Spanish population and it has been shown that areas of life which are important to measure for a given population may not be included in instruments developed in other cultures [17].

In an attempt to overcome some of these drawbacks to measurement of HRQOL of patients with psoriasis in Spain, the PSO-LIFE (Psoriasis Quality of Life) instrument was developed [18]. It is a 20 item instrument covering aspects ranging from symptoms to the impact on emotional well-being, relationships, and activities and leisure, which had not been validated to date.

The objective of the present study was to validate the new questionnaire. More specifically, the aim was to determine its reliability, construct validity, and sensitivity to change.

## Methods

### Study design

This was an observational, prospective, multicenter study to validate a new HRQOL instrument (PSO-LIFE) for patients with psoriasis. Patients were included in the study between October 2008 and May 2009, and attended a maximum of 3 study visits over the 3 month follow-up period. A total of 39 investigators from centers all over Spain participated in the study.

The study protocol was approved by the Clinical Research Ethics Committee of the Hospital General Universitario de Valencia. All patients included in the study provided their signed, informed consent to participate.

### Study population

Three types of patients were included in the study. Patients in Group A were patients with a diagnosis of active psoriasis, patients in Group B had inactive psoriasis at the time of inclusion, and patients in Group C were a control group consisting of patients with active atopic dermatitis (AD) or active chronic urticaria (CU). Inactive psoriasis was defined as occurring when plaques remained the same size and no new plaques appeared, even when psoriasis was widespread [19]. Patients in groups A and B completed 3 study visits, at baseline, and at 7 days and 3 months since baseline. Patients in Group C only attended one study visit.

Inclusion criteria for Group A patients (patients with active psoriasis) were: over 18 years of age with a diagnosis of plaque psoriasis for at least 6 months and confirmed by a dermatologist, who was able to complete the study questionnaires, and who provided written, informed consent to participate. Patients were excluded from this group if they had guttate, erythrodermic or pustular psoriasis, psoriatic arthritis, or psoriasis limited to the scalp, nails, palms, or soles. The same inclusion criteria were applied for Group B, although in that case patients were required to have inactive psoriasis. Patients included in the control group were required to have a confirmed diagnosis of AD or CU.

### Sample size

Sample size was set at 325 patients aged over 18 with a diagnosis of active or inactive psoriasis and 66 patients in the control group (33 with AD and 33 with CU). The sample size was calculated so as to allow for testing a full range of psychometric characteristics and was based specifically on the need to test sensitivity to change which was considered to require the largest sample size. Specifically, sample size for patients groups A and B was calculated to be able to detect a change of 0.2 standard deviations on the PSO-LIFE score after 3 months of follow-up, with a p value of 0.01 and a statistical power of 0.80 assuming a 10% loss to follow-up. Applying the same criteria, and in order to detect a between-group difference of 0.45 SDs with a ratio of inactive patients to controls and active patients to controls of 1:2 and 1:3, respectively, a sample size of 65 controls, 130 patients with inactive psoriasis, and 195 patients with active psoriasis was required.

### Study instrumentation and variables

The primary study variable was the PSO-LIFE questionnaire. This was a self-administered questionnaire for patients with psoriasis consisting of 20 items measuring a range of HRQOL aspects relevant to individuals with psoriasis (Additional file 1). The items refer to the previous 7 days and each item allows for a 5 point Likert scale (response choices from *Always* to *Never*), Each response is given a scoring from 1 (worst HRQOL) to 5 (best HRQOL). The overall questionnaire score is obtained by adding up the 20-item responses and it ranges from 20 to 100 points. In order to simplify its interpretation and to validate the PSO-LIFE questionnaire, the scoring was transformed to a 0 to 100 scale with a higher score indicating better HRQOL.

The PSO-LIFE questionnaire was developed in a previous study phase using questionnaire development techniques; according to FDA guidelines for patient-reported outcome measure [20], a conceptual framework was established for the questionnaire development. Psoriasis is a dermatological disease which affects physical, emotional and social well-being, these conform the HRQOL in patients with psoriasis. The physical dimension is affected through its symptoms; the psychological dimension is affected through anxiety, depression, lack of concentration and altering self-image; and it also affects the social domain through the limitation on social/leisure activities, disturbing sex life and causing work leave. The questionnaire to be developed pretended to consider the relationship among all these domains and their impact on patient’s life. Considering all these aspects, the initial point was the development of a specific questionnaire that would provide physicians with a tool to assess knowledge on the impact of psoriasis on the patient’s quality of life.

Firstly, a literature review was performed to set the relation between psoriasis and patient’s self-perceived quality of life; then, a qualitative assessment through a focus group with 5 dermatologists was carried out to identify the main domains affecting HRQOL in psoriasis patients, As a second step, a semi-structured interview was conducted with 20 psoriasis patients (10 with active and 10 with non active), from which several items were identified to be related with psoriasis HRQOL; at the end of this stage 139 preliminary statements were identified. Each of the remaining items was subsequently rated by the dermatologist in terms of frequency, importance and clarity and the number of items was reduced to a total of 37 items. The selected items were edited in questionnaire format and administered to a sample of 171 psoriasis patients (52.1% active and 47.9% non active) to allow for a preliminary factor analysis and Rasch analysis in order to obtain the final version of the questionnaire. Factor analysis identified 6 preliminary dimensions (variance explained of 72.4%). The Rasch analysis was used to exclude those items with INFIT or OUTFIT > 1.30 and <0.70 and those which were redundant with other items. This final questionnaire, with a total of 20 items has good preliminary internal consistency (Cronbach’s alpha = 0.94) [18].

In the present study, socio-demographic variables collected were age, sex, educational level (no education, primary, secondary or tertiary level studies), and employment situation. Clinical variables collected in patients with psoriasis included date of diagnosis, duration of latest episode of active psoriasis, PASI index, current treatment, recurrence or continuation of active episode (in follow-up visits), and co-morbid chronic diseases. In patients with AD, clinical variables included severity of AD using the EASI index, disease intensity (using a 4 point scale from none to severe), and treatment. In patients with CU, variables collected included type of CU, current symptoms, presence of wheals or lesions on inclusion, and treatment.

As well as the PSO-LIFE questionnaire, all patients also completed the Dermatology Life Quality Index (DLQI) and patients in groups A and B also completed the Psoriasis Disability Index (PDI). Both of these have been adapted and validated for use in Spain [15][16]. The DLQI is a generic questionnaire designed for use in patients with any type of skin disease. It consists of 10 items with a time-frame referring to the last 7 days and measures the impact of disease in terms of symptoms, daily activities, leisure, work/study, personal relationships, and treatment. The score ranges from 0 (minimal impact on HRQOL) to 30 (maximum impact on HRQOL). The PDI is a disease-specific questionnaire consisting of 15 items covering four dimensions of HRQOL: daily activities, work/studies, personal relationships, and leisure, with an additional item on treatment. The overall score ranges from 0 to 45 with a higher score indicating greater impact on HRQOL.

Data were also collected on patient perception of their overall health state using a single item with 7 response options. In the follow-up visits, patients were also asked whether their overall health state had improved, deteriorated or remained the same, again using a 7 option response scale from much improved to much worse (health status transition item).

### Statistical analysis

The data analysis was performed using SPSS 15.0 for Windows. A significance level of 0.05 was used in all between-group comparisons.

The sample’s socio-demographic and clinical characteristics were analyzed using descriptive statistics. Parametric (Student’s *t* test and ANOVA) and non-parametric (Mann-Whitney U, Kruskal-Wallis, chi-squared) tests were used to test for between-group differences while Friedman and Wilcoxon tests for paired data were used to test for differences over time in the PSO-LIFE index.

The feasibility of the PSO-LIFE questionnaire was tested by analyzing the proportion of patients with missing responses on each item, and the proportion of patients with no missing responses.

Score distributions were evaluated by calculating the observed range of scores and the proportion of patients with the worst and best possible scores (floor and ceiling effects) on each dimension, as an indicator of the extent to which scales capture the range of the underlying dimension. The reliability of the new questionnaire was tested by examining the internal consistency of data in the overall sample of psoriasis patients using Cronbach’s α. Test-retest reliability was examined in patients (group A and B) who reported no change on the health status transition item in the follow-up visit at 7 days and was analyzed using the Intraclass Correlation Coefficient (ICC). For both Cronbach’s α and the ICC, values over 0.7 were considered acceptable [21].

Construct validity was tested in several ways. In the first place, a principal components factor analysis was performed with Varimax rotation to determine the underlying dimensional structure of the questionnaire. Screen plot analysis (based on eigenvalues –which correspond to the variances of the factors– was also used in deciding the number of factors in the questionnaire [22].

The questionnaire’s known groups’ validity (or capacity to discriminate between groups expected to have different scores [23]) was assessed by comparing scores on the PSO-LIFE questionnaire between patients with active and inactive psoriasis, and between those two groups and the control group. We also expected patients with visible lesions to score worse on the PSO-LIFE than those without visible lesions, and we expected those with mild psoriasis (PASI score <10) to score higher on the PSO-LIFE (better HRQOL) than those having more severe psoriasis (PASI score of 10 – 50). Between-group differences were analyzed using linear regression models and controlling for age and level of education (patients vs. controls) and age, level of education, and duration of last crisis (active vs. inactive psoriasis).

Convergent validity was tested by examining the extent to which scores on the PSO-LIFE demonstrated logical relationships with the DLQI and PDI in the baseline visit. We expected moderate to high correlations with the DLQI and PDI overall scores and with most of the individual dimensions on those questionnaires as they measure similar content to the PSO-LIFE. Correlations were calculated using Pearson and Spearman correlation coefficients as appropriate. Correlations under 0.3 were considered weak, correlations between 0.3 and 0.5 were considered moderate, and those over 0.5 were considered strong [24].

Longitudinal validity and sensitivity to change were examined by comparing changes observed in PSO-LIFE scores with those observed on the PASI Index, psoriasis status, changes in treatment, and presence of adverse effects as well as with changes in scores on the DLQI and PDI, between baseline and the final visit. Changes in PSO-LIFE score were analyzed by sub-groups based on patient perceptions of overall change on completing the study using the health status transition item. Effect sizes were used to estimate the magnitude of changes. Effect size was defined as the difference between baseline and final visit mean scores divided by standard deviation at baseline [24]. An effect size of 0.2 is equivalent to a small effect size, 0.5 equates to a medium effect size, and values of 0.8 or over equate to large effect sizes [24]. For comparison, effect sizes for the DLQI and PDI questionnaires were also calculated.

The minimum important difference (MID) is the smallest difference in scores on a questionnaire that patients perceive as beneficial [25]. The MID was estimated for the PSO-LIFE questionnaire using results from patients who reported having 'slightly improved' health status at 3 months from baseline.

## Results

The final sample included for analysis consisted of 304 patients and 56 controls. Of the patients, 182 (50.6%) were patients with active psoriasis and 122 (33.9%) had inactive psoriasis, which was in accordance with the initially expected sample size, considering a 10% follow up. Of the controls, 26 (7.2%) were patients with CU and 30 (8.3%) were patients with AD. Sample socio-demographic and clinical characteristics at baseline are shown in Table 1. The mean (SD) age of psoriasis patients was 45.3 (14.5) years compared to 38.8 (14) years for controls (p < 0.01). In the psoriasis group, 56.3% of patients were men compared to 51.8% in controls (p = 0.54), and the control group showed higher levels of education (46% who had completed university level education compared to 24% of patients; p < 0.01). In terms of clinical characteristics, patients reported a mean of 18 years with the symptoms. The mean (SD) PASI Index score at baseline was 17 (7.4) in patients with active psoriasis and 5.6 (5.3) in patients with inactive psoriasis (p < 0.01).

**Table 1:** Sample characteristics at baseline: controls, patients with active and inactive psoriasis

	**Patients (active psoriasis)N = 182**	**Patients (inactive psoriasis)N = 122**	**Controls N = 56**	P**
***Socio-demographic variables***				
Age, mean (SD), years	43.6 (14.7)	47.8 (14.0)	38.8 (14.0)	<0.01
Male, n (%)	101 (55.5)	70 (57.4)	29 (51.8)	0.54
Highest educational level, n (%)				<0.01
*No formal education*	5 (2.8)	4 (3.3)	4 (7.1)	
*Primary*	66 (36.9)	46 (38.0)	11 (19.6)	
*Secondary*	62 (34.6)	45 (37.2)	15 (26.8)	
*Post-secondary*	46 (25.7)	26 (21.5)	26 (46.4)	
Employment status, n (%)				<0.05
*Unemployed*	19 (10.4)	12 (9.8)	6 (10.7)	
*Working – self-employed*	26 (14.3)	11 (9.0)	10 (17.9)	
*Working – employed*	87 (47.8)	59 (48.4)	28 (50.0)	
*Work disability*	1 (0.5)	4 (3.3)	1 (1.8)	
*Pensioner/retired*	19 (10.4)	25 (20.5)	3 (5.4)	
*Looking after home*	22 (12.1)	9 (7.4)	7 (12.5)	
*Student*	9 (4.9)	5 (4.1)	7 (12.5)	
***Clinical variables***				
Years since diagnosis, mean (SD)	18.6 (12.4)	17.8 (11.9)		NS
PASI, n (%)				<0.01
*Mild (<10)*	31 (17.0%)	98 (80.3%)		
*Moderate/severe* ≥ *10*	151 (83.0%)	24 (19.7%)		
Treatment*				
*Any*	177 (97.3%)	104 (85.2%)		<0.01
*Topical*	90 (49.5%)	44 (36.1%)		<0.05
*PUVA/Phototherapy*	25 (13.7%)	10 (8.2%)		NS
*Systemic*	101(55.5%)	54 (44.3%)		NS
*Biological*	57 (31.3%)	59 (48.4%)		<0.01
*Other*	32 (17.6%)	9 (7.4%)		0.01

PASI: Psoriasis Area Severity Index.

*Multi-response. The same patient could be in more than one category. Calculation performed for N = 304 (all patients).

**Statistically significant differences between patients and controls in proportion of Pensioners/retired.

### Feasibility

In terms of feasibility, the items with the highest rate of missing responses were items 15 and 16 which dealt with problems in work/study. In this case, the highest rate of non-response was among pensioners and housewives (<20% who failed to answer). Nevertheless, the entire questionnaire was completed by over 95% of patients, and therefore showed good feasibility (Table 2).

**Table 2:** Distribution characteristics and reliability of the PSO-LIFE questionnaire in psoriasis patients (active and inactive): n = 304

	**Mean**	**SD**	Missing items*	**Observed range**	**Floor (%)**	**Ceiling (%)**	**Cronbach’s alpha**	**ICC**
PSO-LIFE overall score	64.9	22.4	4.6%	10-100	0%	3.2%	0.95	0.98

*Proportion of patients with a missing response on at least one item.

### Reliability

No study patients obtained the minimum PSO-LIFE score (floor effect) and only 3.2% obtained the maximum score (ceiling effect). Reliability assessed in terms of internal consistency was excellent, with a Cronbach’s alpha value of 0.948. Test-retest reliability, which was assessed in patients who remained stable between baseline and follow-up after 7 days (n = 149), was also excellent with an ICC of 0.98 (Table 2).

### Validity

**Figure 1:**
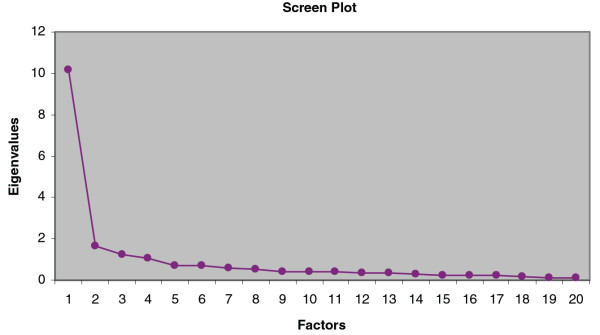
Screen plot analysis of factors in the PSO-LIFE questionnaire.

Factor analysis showed the questionnaire to be essentially unidimensional, with a single dimension explaining 50.9% of the variance in overall score. Screen plot analysis (Figure 1) also indicated a clear threshold of one factor when distinguishing between factors with high and low eigenvalues.

When PSO-LIFE scores were compared between patients with psoriasis and controls, statistically significant differences were observed (p = 0.03, adjusted by age and education) (Table 3). The difference in scores between those with active and inactive psoriasis was also notable, with mean (SD) scores of 57.4 (20.4) and 76.4 (20.6), respectively (p < 0.01, adjusted by age, education and duration of last outburst). On the other hand, statistically significant differences were not observed between patients with inactive disease and the control group (p > 0.05). Scores on the PSO-LIFE also varied according to disease localization. Patients with visible lesions (for example, on the head or upper extremities) had a mean (SD) score of 63 (22) compared to 74.8 (23.9) in patients with non-visible lesions and 78.5 (21.6) in patients without lesions in the baseline visit (p < 0.01). Finally, statistically significant differences were also observed between patients with mild disease and those with moderate disease defined using the PASI (p < 0.01) (see Table 3).

**Table 3:** Known groups’ validity: PSO-LIFE scores according to patient type, psoriasis status, localization, and disease severity

**Groups**	**Mean PSO-LIFE score (SD)**	**P value**
Patient type		0.03
*Psoriasis, n = 304*	64.9 (22.5)	
*Control, n = 56*	69.4 (17.3)	
Psoriasis status		<0.01
*Active, n = 182*	57.4 (20.4)	
*Inactive, n = 122*	76.4 (20.6)	
Localization		<0.01
*No lesions, n = 25*	78.5 (21.6)	
*Trunk, lower extremities, n = 14*	74.8 (23.9)	
*Head, upper extremities, n = 251*	63.0 (21.9)	
Psoriasis severity		<0.01
*Mild (PASI <10), n = 124*	74.5 (22.0)	
*Moderate/severe ≥ 10, n = 166*	57.8 (20.1)	

In comparison with the other disease-specific instruments used, the standardized differences in PSO-LIFE scores between patients with active and inactive disease were larger than the differences found on the DLQI and PDI (standardized differences of 0.85 standard deviations on the PSO-LIFE compared to 0.79 on the DLQI and 0.62 on the PDI).

The correlation between PSO-LIFE and PASI scores was moderate (r = −0.43) while correlations with the DLQI and PDI ranged from moderate to high (between −0.4 and −0.8 in the different questionnaires). The correlation with the DLQI global score was r = −0.76 (p < 0.01) and with the PDI overall score r = −0.76 (p < 0.01). The highest (negative) correlation coefficients between the PSO-LIFE and the DLQI were observed on the symptoms and perceptions dimension (r = −0.78, p < 0.01), followed by activities of daily living and leisure (r = −0.66, p < 0.01). The lowest correlation was seen with the DLQI work and study dimension (r = −0.40, p < 0.01). With the PDI, high correlations were observed with daily activities and leisure dimensions (r = −0.72 and −0.66, respectively; p < 0.01). Overall, correlations were slightly higher with the DLQI than with the PDI.

### Longitudinal validity and sensitivity to change

The correlation coefficient between changes observed on the PSO-LIFE and changes observed on the PASI index between baseline and the final visit was r = −0.4 (p < 0.01). Patients with active disease at baseline but who were inactive at study completion showed a mean (SD) improvement in PSO-LIFE score of 19.9 (15.1), while patients who still had active disease at the end of the study improved by 10.1 (15.8) points. Patients who had inactive disease throughout the study improved by 7.7 (15.7) points, whilst those who became active during the study showed a worsening in score of 8.2 (14.9) points (p < 0.01). On the other hand, no differences in score changes were observed according to whether the patients required a change in treatment or not (p = 0.94) or if they presented an adverse event during the study (p = 0.47), as these could occur at any time during follow-up and the questionnaire measures HRQOL during the last 7 days. Changes in score on the PSO-LIFE showed moderate to high correlations with changes in score on the DLQI (r = −0.69) and PDI (r = −0.67) questionnaires.

The effect size for the PSO-LIFE questionnaire in patients who reported a ‘much improved’ health state on completing the study was 1.01 (large effect size) (Table 4). The effect size decreased as the reported overall change in health status diminished and, in patients reporting an identical or very similar health state at the end of the study, the effect size was 0.15 (small or null effect size). The effect size for the sample that completed the study was 0.5 (medium effect size), 0.73 for patients with active psoriasis, and 0.29 for patients with inactive psoriasis. Effect sizes for the DLQI and PDI were smaller than for PSO-LIFE at 0.44 and 0.39, respectively.

**Table 4:** PSO-LIFE scores at baseline and final visit, difference in score between visits, and effect size according to change in psoriasis patients’ self-perceived health status

**Self-rating on health status transition item**		**Baseline**		**Final (3 months)**		**Difference**		**n**	**Effect size**
		**Mean**	**SD**	**Mean**	**SD**	**Mean**	**SD**		
Much improved		62.3	22.0	84.5	13.4	22.2	20.4	69	1.01
Considerably improved		58.0	21.0	75.0	17.6	17.1	12.8	67	0.81
Slightly improved		68.5	22.6	75.0	20.1	6.5	10.3	39	0.29
More or less the same		73.5	20.6	76.5	20.6	3.0	9.8	62	0.15
Slightly worse		68.4	24.6	62.3	22.4	−6.2	15.5	16	−0.25
Considerably/Much worse		63.0	17.5	55.2	17.4	−7.7	15.1	11	−0.44
	TOTAL	65.1	22.1	76.2	19.2	11.1	17.4	264	0.50

**Figure 2:**
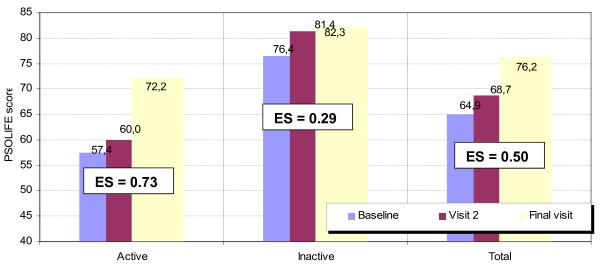
Change in PSO-LIFE scores over the 3 month study period according to baseline group (active or non-active disease).

The largest changes in PSO-LIFE scores over time were seen for the group with active psoriasis at baseline. Change over time was notably smaller in the inactive disease group (see Figure 2).

The MID at 3 months was approximately 6.5 points.

### Recommendations for PSO-LIFE questionnaire utilization

The PSO-LIFE questionnaire can also be used in clinical practice obtaining the direct scores and without having to transform them to a 0 to 100 scale. Using the score obtained from a direct sum of items (ranging from 20 to 100) the MID at 3 months was approximately 5.2 points. The transformation of scores does not affect the questionnaire validity, therefore, even though the validity process was done using the transformed values, the direct scores can also be used.

In terms of imputation to missing values, no assumption of minimum requested items answered has been made due to the low number of missing responses in the validation study. Nevertheless, the authors recommend that the number of missing items shouldn't be higher than 25%, which corresponds to a maximum of 5 missing items in the PSO-LIFE questionnaire. In case of missing items, they are given the value which is the mean of the rest of the items which have been answered by the patient.

## Discussion

Numerous studies have shown that psoriasis has a negative impact on the quality of life of patients and family members [3]. However, existing instruments have been shown to have some drawbacks and none of them were developed in Spain. This study has shown that the PSO-LIFE questionnaire, a new tool for measuring HRQOL in patients with psoriasis has good acceptability and ease of administration together with excellent indices of reliability, validity, and sensitivity to change. The PSO-LIFE adds a further option in the range of instruments available to measure HRQOL in patients with psoriasis.

The fact that over 95% of patients completed all of the items of the questionnaire suggests that the questionnaire is feasible, and may also suggest that the items are generally relevant to patients. Interestingly, the items with the highest rate of missing responses were the items on the impact of the disease on work and study, where the highest rate of missing responses were observed among pensioners and students. A higher rate of missing responses among the first group on these items might be expected as they could consider the items not relevant to them, though it is not so clear why students should consider the items non-relevant. On the other hand, even in these groups the rate of missing responses was not too high, and we consider it essential to maintain these items, given their relevance among psoriasis sufferers in general [26].

Reliability coefficients, both in terms of internal consistency and test-retest reliability were extremely high, and easily exceeded the threshold of 0.70 suggested as an indicator of satisfactory levels of reliability in this type of instrument [21]. The results in terms of Cronbach’s alpha compare favorably to coefficients observed for psoriasis-specific outcomes such as the Psoriasis Symptom Assessment (PSA) [27] or the PSORIQOL as well as to dermatology-related instruments such as the DLQI [27].

In terms of validity, we performed a wide range of tests which generally indicated acceptable validity. Factor analysis showed that the questionnaire was essentially unidimensional. The undimensionality reflects the diversity of the questions conforming this questionnaire, this heterogeneity might be explained because of the short number of items considered in the questionnaire (only 20) and the high number of factors that might affect somehow the HRQOL in psoriasis patients; actually the 20 items assess symptomatology, emotional well-being, personal and social relationships, as well as leisure.

Nevertheless the unidimensionality has some disadvantages, as it means that scores are not available for individual HRQOL dimensions, which might be of interest from a clinical point of view. On the other hand, the unidimensionality likely contributes to achieving the high reliability coefficients observed here, which means that users can have substantial confidence in the scores obtained. Likewise, the fact that the instrument is unidimensional will facilitate scoring and the interpretation of scores. When using multidimensional instruments, patients may improve on some dimensions and worsen on others, making it difficult to assess the overall direction of change.

Apart from the wide range of tests employed to examine the validity of the new instrument, other strengths of the present study included the involvement of patients with active and inactive disease, the use of control groups, and testing of the sensitivity to change. The first and last of these are related and were of considerable relevance in this study as we were particularly interested in developing and testing an instrument that would discriminate well across the spectrum of disease, and which would show good responsiveness, an aspect which has not been determined for some available instruments [28] or which has occasionally been shown to be unsatisfactory [1]. Here, the PSO-LIFE questionnaire showed good responsiveness and was noticeably more sensitive to change than the DLQI or the PDI.

It is well known that some of the questions contained in the existing questionnaires do not really fit within the Spanish cultural way of living, as they have been developed with anglo-saxon patients. The fact that PSO-LIFE has been developed in Spain makes it a very valid alternative, or even more appropriate, than the current existing questionnaires in Spain, as well as in other Mediterranean countries where the culture of the patients is similar to Spain.

On the other hand, one of the limitations of the study was that sensitivity to change was not tested in a before and after design, i.e. by administering the test before and after an intervention of known efficacy. Instead, we used the approach of determining whether the instrument was responsive to changes in patient’s self-assessed overall health status. This approach has some limitations [29] but is widely used and provides at least provisional evidence for the instrument’s sensitivity. Nevertheless, future studies should test the instrument in a before and after design. Another limitation of the study was that it was only performed in patients with plaque psoriasis. Although this was the main population of interest here and it is the most frequent type of psoriasis, it would nevertheless be useful to test the instrument in patients with other types of psoriasis. Although representativeness is not an essential characteristic for this type of validation study, which requires instead variety particularly in clinical characteristics, a considerable number of centers participated from different regions in Spain in order to collect the variability in terms of clinical practice.

## Conclusions

We believe that the present results provide substantial support for the reliability, validity, and responsiveness of the PSO-LIFE questionnaire in the population for which it was designed. There is also preliminary evidence for superior discriminatory capacity and sensitivity to change than other questionnaires frequently used in these patients, i.e. the DLQI and the PDI. Further studies are required to assess the instrument’s responsiveness in a before and after setting with an intervention of known clinical efficacy and to test the instrument in patients with other types of psoriasis. Adaptation and validation of the instrument in other languages and settings would also be useful in determining its potential for use in multinational studies and for comparing the HRQOL of psoriasis patients in different countries.

## Competing interests

Esteban Dauden: Advisory Board member, consultant, grants, research support, participation in clinical trials, honorarium for speaking, research support, with the following pharmaceutical companies: Abbott, Amgen, Astellas, Centocor Ortho Biotech Inc., Galderma, Glaxo, Janssen-Cilag, Leo Pharma, Novartis, Pfizer, MSD, Celgene. Enrique Herrera: None declared. Lluis Puig: Has received from Merck honorarium for speaking at meetings, participation on advisory boards and participation in clinical trials. José Luis Sánchez-Carazo: None declared. Jaime Toribio: None declared. Ma. Teresa Caloto: None declared. Gonzalo Nocea: None declared. Montse Roset: None declared. Nuria Lara: None declared.

## Author’s contributions

ED, EH, LlP, JLSC, JT, MR y NL conceptualized the study and developed the questionnaire. ED, EH, LlP, JLSC and JT obtained the data. ED, EH, LlP, JLSC, JT, MR y NL analyzed the data. All authors provided input on the interpretation and they read and approved of the final draft of the manuscript.

## Supplementary Material

Additional file 1 PSO-LIFE Questionnaire. Click here for file
