# Supplementary material for: Validation of a new tool to assess health-related quality of life in psoriasis: the PSO-LIFE questionnaire
Source: Health Qual Life Outcomes. 2012 May 24;10:56. doi: 10.1186/1477-7525-10-56 (PMC3458920; doi:10.1186/1477-7525-10-56)
Supplement: Additional file 1 — PSO-LIFE Questionnaire. [file 1477-7525-10-56-S1.doc]

PSO-LIFE Questionnaire

Think about how you feel about your psoriasis over the last seven days and mark with an ‘X’ the response that best defines your situation:

1. The **burning** that I notice in my skin is very unpleasant.
2. The **itching** in my skin causes me to scratch myself and to cause myself to bleed.
3. The **itching** in my skin is uncontrollable and prevents me from concentrating on what I am doing when it occurs.
4. My **nails** show **changes** and are painful.
5. The appearance of my skin **saddens** me.
6. It **embarrasses** me to feel I am being looked at.

1. I feel **unwanted** by other people because of the lesions on my skin.
2. Having psoriasis negatively affects my **relationships with other people**.
3. I am always waiting for changes in the number or extension of the lesions on my skin.
4. Having psoriasis causes me to be more **withdrawn** and to be more reserved.
5. Having psoriasis causes me to feel less self confident.
6. It **depresses** me (I feel down) to think that psoriasis is a life-long condition.
7. I try to hide the changes on my nails and hands so that other people can’t see them.
8. I avoid going to the swimming pool/beach because it **depresses** me when I notice that people move away from me when they see the lesions on my skin.
9. I have **problems at work/in my place of study** because the lesions on my skin are visible and cause a negative reaction from the people around me.
10. I am **stressed at work/in my place of study** because I try to hide the lesions on my skin from other people.
11. I prefer to be at home and **do not have interest in meeting other people**, so that other people do not see me.
12. It **worries** me to think that psoriasis can worsen.
13. It **embarrasses** me to undress in front of a person with whom I am going to have sexual relations.
14. The lesions on my skin are the main reason for my **rejection** of intimate relationships
